# A Suggestion

**Published:** 1877-09

**Authors:** 


### A SUGGESTION.

A North Carolinian, an old subscriber, whose heart was always big as the world, and whose business energy and integrity have few superiors, writes: "Doctor, I have a request to make and a large one, too. I don't know as I could do better than to have a few thousands expended in sending your JOURNAL all over the country wherever men and women can read; the thousands of people who never see your publications are the very ones who would most appreciate such a privilege; they are the poorer classes, the people who could understand what you say, and thereby be elevated into a higher sphere of life. In my opinion your writings will grow more and more in public favor and outlive the cart loads of stuff, called literature, so cheaply retailed by news-venders now-a-days. Go on, Doctor; you are on the right track and have lived too long to be thrown off."

To which we reply, Only the intelligent and thinking few can be induced to patronize what is true, practical and useful. Health, like religion, is never placed at its real value until the opportunity of securing it is gone forever. Millions of money are spent every month in the purchase of transient and trashy novels; all classes join in this expenditure, and yet, when a dollar or two would purchase reading, for an entire family for a whole year, which shows how to maintain health, and how to avert disease, bodily, mental, and moral, for a life-time, the expenditure is considered one of the things which can be dispensed with without inconvenience. So much the better for doctors, who profit by the negligence or stupidity to the amount of a hundred million of dollars every year in the United States alone, besides another hundred million to druggists and more than another hundred million for quack medicines, such as molasses and water, opium, colored soap-suds, and the like. Next to the promotion of religion and education, we do not know a greater good could be done, at so small an expense.

